# Methyl 2-(5-bromo-3-methyl­sulfinyl-1-benzofuran-2-yl)acetate

**DOI:** 10.1107/S1600536808037768

**Published:** 2008-11-20

**Authors:** Hong Dae Choi, Pil Ja Seo, Byeng Wha Son, Uk Lee

**Affiliations:** a Department of Chemistry, Dongeui University, San 24 Kaya-dong, Busanjin-gu, Busan 614-714, Republic of Korea; b Department of Chemistry, Pukyong National University, 599-1 Daeyeon 3-dong, Nam-gu, Busan 608-737, Republic of Korea

## Abstract

The title compound, C_12_H_11_BrO_4_S, was synthesized by the oxidation of methyl 2-(5-bromo-3-methyl­sulfanyl-1-benzofuran-2-yl)acetate with 3-chloro­peroxy­benzoic acid. The O atom and the methyl group of the methyl­sulfinyl substituent lie on opposite sides of the plane of the benzofuran ring system. The crystal structure is stabilized by C—H⋯π inter­actions, involving a methyl H atom and the benzene ring of a neighbouring mol­ecule, and by weak inter­molecular C—H⋯O hydrogen bonds.

## Related literature

For the crystal structures of similar methyl 2-(3-methyl­sulfinyl-1-benzofuran-2-yl)acetate derivatives, see: Choi *et al.* (2008*a* ▶,*b* ▶).
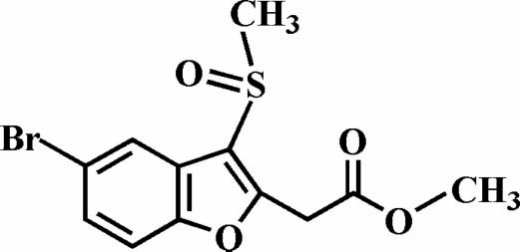


## Experimental

### 

#### Crystal data

C_12_H_11_BrO_4_S
*M* *_r_* = 331.18
Triclinic, 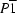

*a* = 7.9696 (5) Å
*b* = 9.1146 (6) Å
*c* = 10.3100 (7) Å
α = 72.587 (1)°
β = 78.716 (1)°
γ = 69.082 (1)°
*V* = 664.17 (8) Å^3^
*Z* = 2
Mo *K*α radiation
μ = 3.25 mm^−1^
*T* = 298 (2) K
0.40 × 0.30 × 0.20 mm

#### Data collection

Bruker SMART CCD diffractometer
Absorption correction: multi-scan (*SADABS*; Sheldrick, 1999 ▶) *T*_min_ = 0.327, *T*_max_ = 0.530
3786 measured reflections
2560 independent reflections
2084 reflections with *I* > 2σ(*I*)
*R*_int_ = 0.015

#### Refinement

*R*[*F* ^2^ > 2σ(*F* ^2^)] = 0.035
*wR*(*F* ^2^) = 0.099
*S* = 0.98
2560 reflections
164 parameters
H-atom parameters constrained
Δρ_max_ = 0.35 e Å^−3^
Δρ_min_ = −0.80 e Å^−3^

### 

Data collection: *SMART* (Bruker, 2001 ▶); cell refinement: *SAINT* (Bruker, 2001 ▶); data reduction: *SAINT*; program(s) used to solve structure: *SHELXS97* (Sheldrick, 2008 ▶); program(s) used to refine structure: *SHELXL97* (Sheldrick, 2008 ▶); molecular graphics: *ORTEP-3* (Farrugia, 1997 ▶) and *DIAMOND* (Brandenburg, 1998 ▶); software used to prepare material for publication: *SHELXL97*.

## Supplementary Material

Crystal structure: contains datablocks I. DOI: 10.1107/S1600536808037768/su2077sup1.cif

Structure factors: contains datablocks I. DOI: 10.1107/S1600536808037768/su2077Isup2.hkl

Additional supplementary materials:  crystallographic information; 3D view; checkCIF report

## Figures and Tables

**Table 1:** Hydrogen-bond geometry (Å, °) *Cg* is the centroid of the benzene ring C2–C7.

*D*—H⋯*A*	*D*—H	H⋯*A*	*D*⋯*A*	*D*—H⋯*A*
C3—H3⋯O2^i^	0.93	2.43	3.334 (4)	163
C9—H9*B*⋯O1^ii^	0.97	2.59	3.555 (3)	171
C9—H9*A*⋯O2^iii^	0.97	2.22	3.177 (4)	169
C12—H12*B*⋯*Cg*^iv^	0.96	2.99	3.903 (4)	159

Symmetry codes: (i) 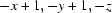; (ii) 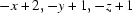; (iii) 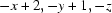; (iv) 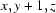.

**Fig. 1.**
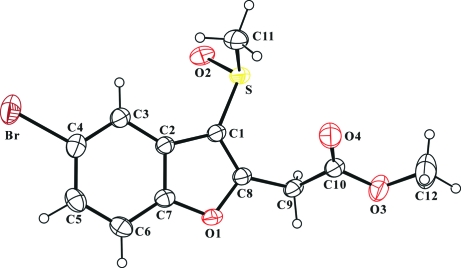
The molecular structure of compound (I), showing the atom numbering scheme and displacement ellipsoids drawn at the 30% probability level.

**Fig. 2.**
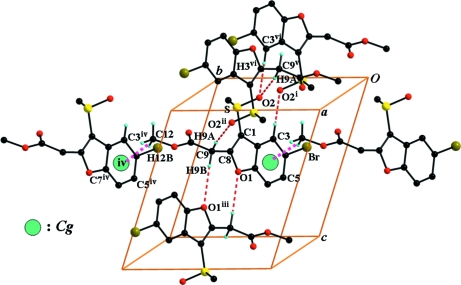
A view of the crystal packing of compound (I), showing the C—H···π and C—H···O interactions as dotted lines. Cg denotes the ring centroid. [Symmetry codes: (i) -x + 1, -y + 1, -zz; (ii) -x + 2, -y + 1, -z + 1; (iii) -x + 2, -y + 1, -z; (iv) x, y + 1, z; (v) -x + 2, -y + 1, -z; (vi) -x + 1, -y + 1, -z.]

## supplementary crystallographic information

### Comment

As a part of our ongoing research on the synthesis and structure of methyl 2-(3-methylsulfinyl-1-benzofuran-2-yl)acetate analogues, the crystal structure of methyl 2-(5-methyl-3-methylsulfinyl-1-benzofuran-2-yl)acetate (Choi *et al*., 2008*a*) and methyl 2-(5-chloro-3-methylsulfinyl-1-benzofuran-2-yl) acetate (Choi *et al.*, 2008*b*) have been reported. Here we describe the crystal structure of the title compound (I), synthesized by the oxidation of methyl 2-(5-bromo-3-methylsulfanyl-1-benzofuran-2-yl)acetate with 3-chloroperoxybenzoic acid.

The molecular structure of compound (I) is illustrated in Fig. 1. The benzofuran unit is essentially planar, with a mean deviation of 0.013 (2) Å from the least-squares plane defined by the nine constituent atoms.

The crystal packing of compound (I) (see Fig. 2) is stabilized by intermolecular C—H···π interactions between an H-atom of the C12-methyl group and the benzene ring of the benzofuran fragment, with a C12—H12B···*Cg*^iv^ separation of 3.903 (4) Å (Fig. 2 and Table 1; *Cg* is the centroid of benzene ring C2–C7, symmetry codes as in Fig. 2). In addition, the molecular packing exhibits three intermolecular C—H···O hydrogen bonds (Fig. 2 & Table 1).

### Experimental

77% 3-Chloroperoxybenzoic acid (190 mg, 0.85 mmol) was added in small portions to a stirred solution of methyl 2-(5-bromo-3-methylsulfanyl-1-benzofuran-2-yl)acetate (252 mg, 0.8 mmol) in dichloromethane (30 ml) at 273 K. After stirring for 3 h at rt, the mixture was washed with a saturated sodium bicarbonate solution and the organic layer was separated, dried over magnesium sulfate, filtered and concentrated under vacuum. The residue was purified by column chromatography (hexane-ethyl acetate 1:2, *v*/*v*) to afford compound (I) as a colorless solid [yield 86%, m.p. 405–406 K; *R*_f_ = 0.45 (hexane-ethyl acetate, 1:2, *v*/*v*)]. Single crystals, suitable for X-ray analysis, were prepared by evaporation of a solution of compound (I) in benzene at rt. Spectroscopic analysis: ^1^H NMR (CDCl_3_, 400 MHz) δ 3.06 (s, 3H), 3.75 (s, 3H), 4.04 (s, 2H), 7.34–7.53 (m, 2H), 7.44 (d, J = 8.80 Hz, 1H), 8.07 (d, J = 1.84 Hz, 1H); EI—MS 332 [*M*+2], 330[*M*^+^].

### Refinement

All the H-atoms were geometrically positioned and refined using a riding model: C—H = 0.93 (aromatic), 0.97 (methylene), and 0.96 Å (methyl) H atoms, with *U*iso(H) = 1.2Ueq(C) (aromatic & methylene), and 1.5Ueq(C) (methyl) H atoms.

### Crystal data

**Table:**

C_12_H_11_BrO_4_S	*Z* = 2
*M**_r_* = 331.18	*F*_000_ = 332
Triclinic, *P*1	*D*_x_ = 1.656 Mg m^−^^3^
Hall symbol: -P 1	Melting point = 405–406 K
*a* = 7.9696 (5) Å	Mo *K*α radiation λ = 0.71073 Å
*b* = 9.1146 (6) Å	Cell parameters from 1892 reflections
*c* = 10.3100 (7) Å	θ = 2.1–27.8º
α = 72.587 (1)º	µ = 3.25 mm^−^^1^
β = 78.716 (1)º	*T* = 298 (2) K
γ = 69.082 (1)º	Block, colourless
*V* = 664.17 (8) Å^3^	0.40 × 0.30 × 0.20 mm

### Data collection

**Table:**

Bruker SMART CCD diffractometer	2560 independent reflections
Radiation source: fine-focus sealed tube	2084 reflections with *I* > 2σ(*I*)
Monochromator: graphite	*R*_int_ = 0.015
Detector resolution: 10.0 pixels mm^-1^	θ_max_ = 26.0º
*T* = 298(2) K	θ_min_ = 2.5º
φ and ω scans	*h* = −9→5
Absorption correction: multi-scan(SADABS; Sheldrick, 1999)	*k* = −11→11
*T*_min_ = 0.327, *T*_max_ = 0.530	*l* = −12→12
3786 measured reflections	

### Refinement

**Table:**

Refinement on *F*^2^	Secondary atom site location: difference Fourier map
Least-squares matrix: full	Hydrogen site location: inferred from neighbouring sites
*R*[*F*^2^ > 2σ(*F*^2^)] = 0.035	H-atom parameters constrained
*wR*(*F*^2^) = 0.099	*w* = 1/[σ^2^(*F*_o_^2^) + (0.0506*P*)^2^ + 0.5362*P*] where *P* = (*F*_o_^2^ + 2*F*_c_^2^)/3
*S* = 0.98	(Δ/σ)_max_ = 0.001
2560 reflections	Δρ_max_ = 0.35 e Å^−^^3^
164 parameters	Δρ_min_ = −0.80 e Å^−^^3^
Primary atom site location: structure-invariant direct methods	Extinction correction: none

### Special details

**Table:**

Geometry. All e.s.d.'s (except the e.s.d. in the dihedral angle between two l.s. planes) are estimated using the full covariance matrix. The cell e.s.d.'s are taken into account individually in the estimation of e.s.d.'s in distances, angles and torsion angles; correlations between e.s.d.'s in cell parameters are only used when they are defined by crystal symmetry. An approximate (isotropic) treatment of cell e.s.d.'s is used for estimating e.s.d.'s involving l.s. planes.
Refinement. Refinement of *F*^2^ against ALL reflections. The weighted *R*-factor *wR* and goodness of fit *S* are based on *F*^2^, conventional *R*-factors *R* are based on *F*, with *F* set to zero for negative *F*^2^. The threshold expression of *F*^2^ > σ(*F*^2^) is used only for calculating *R*-factors(gt) *etc*. and is not relevant to the choice of reflections for refinement. *R*-factors based on *F*^2^ are statistically about twice as large as those based on *F*, and *R*- factors based on ALL data will be even larger.

### Fractional atomic coordinates and isotropic or equivalent isotropic displacement parameters (Å^2^)

**Table:**

	*x*	*y*	*z*	*U*_iso_*/*U*_eq_	
Br	0.26775 (5)	0.20066 (4)	0.38327 (5)	0.07396 (18)	
S	0.73771 (11)	0.63026 (10)	0.03527 (7)	0.0505 (2)	
O1	0.8367 (3)	0.4636 (2)	0.42105 (19)	0.0450 (5)	
O2	0.7437 (4)	0.4993 (3)	−0.0262 (2)	0.0668 (7)	
O3	1.0188 (3)	0.8974 (3)	0.2240 (3)	0.0718 (7)	
O4	0.7513 (3)	0.8989 (3)	0.1905 (3)	0.0724 (7)	
C1	0.7429 (4)	0.5435 (3)	0.2117 (3)	0.0422 (6)	
C2	0.6455 (4)	0.4363 (3)	0.2987 (3)	0.0408 (6)	
C3	0.5121 (4)	0.3798 (3)	0.2824 (3)	0.0466 (7)	
H3	0.4651	0.4089	0.1993	0.056*	
C4	0.4537 (4)	0.2784 (4)	0.3965 (4)	0.0510 (7)	
C5	0.5227 (5)	0.2305 (4)	0.5228 (4)	0.0550 (8)	
H5	0.4796	0.1604	0.5964	0.066*	
C6	0.6542 (4)	0.2870 (4)	0.5381 (3)	0.0526 (8)	
H6	0.7023	0.2566	0.6210	0.063*	
C7	0.7117 (4)	0.3904 (3)	0.4254 (3)	0.0419 (6)	
C8	0.8535 (4)	0.5552 (3)	0.2893 (3)	0.0413 (6)	
C9	0.9848 (4)	0.6447 (4)	0.2609 (3)	0.0473 (7)	
H9A	1.0742	0.6117	0.1877	0.057*	
H9B	1.0470	0.6145	0.3416	0.057*	
C10	0.9012 (4)	0.8259 (4)	0.2215 (3)	0.0483 (7)	
C11	0.5088 (5)	0.7628 (4)	0.0366 (4)	0.0637 (9)	
H11A	0.4834	0.8234	−0.0551	0.096*	
H11B	0.4918	0.8363	0.0916	0.096*	
H11C	0.4285	0.6997	0.0740	0.096*	
C12	0.9545 (7)	1.0738 (5)	0.1880 (7)	0.1065 (18)	
H12A	1.0493	1.1133	0.1927	0.160*	
H12B	0.8527	1.1114	0.2506	0.160*	
H12C	0.9191	1.1131	0.0968	0.160*	

### Atomic displacement parameters (Å^2^)

**Table:**

	*U*^11^	*U*^22^	*U*^33^	*U*^12^	*U*^13^	*U*^23^
Br	0.0572 (2)	0.0513 (2)	0.1163 (4)	−0.02436 (17)	−0.0138 (2)	−0.0133 (2)
S	0.0535 (5)	0.0626 (5)	0.0392 (4)	−0.0239 (4)	−0.0121 (3)	−0.0067 (3)
O1	0.0450 (11)	0.0494 (11)	0.0411 (10)	−0.0113 (9)	−0.0157 (9)	−0.0094 (9)
O2	0.0746 (17)	0.0842 (17)	0.0506 (13)	−0.0229 (14)	−0.0132 (12)	−0.0286 (12)
O3	0.0615 (15)	0.0579 (14)	0.108 (2)	−0.0253 (12)	−0.0235 (14)	−0.0201 (14)
O4	0.0561 (15)	0.0537 (14)	0.103 (2)	−0.0180 (12)	−0.0308 (14)	0.0016 (13)
C1	0.0433 (15)	0.0454 (15)	0.0412 (14)	−0.0144 (13)	−0.0090 (12)	−0.0122 (12)
C2	0.0431 (15)	0.0361 (14)	0.0437 (15)	−0.0096 (12)	−0.0107 (12)	−0.0105 (12)
C3	0.0475 (17)	0.0398 (15)	0.0533 (17)	−0.0105 (13)	−0.0115 (13)	−0.0131 (13)
C4	0.0404 (16)	0.0385 (15)	0.073 (2)	−0.0091 (13)	−0.0074 (15)	−0.0153 (15)
C5	0.0507 (18)	0.0400 (16)	0.0600 (19)	−0.0085 (14)	−0.0028 (15)	−0.0010 (14)
C6	0.0524 (18)	0.0459 (17)	0.0472 (17)	−0.0063 (14)	−0.0094 (14)	−0.0025 (13)
C7	0.0393 (15)	0.0379 (14)	0.0455 (15)	−0.0044 (12)	−0.0102 (12)	−0.0117 (12)
C8	0.0424 (16)	0.0395 (14)	0.0429 (15)	−0.0099 (12)	−0.0103 (12)	−0.0116 (12)
C9	0.0423 (16)	0.0534 (17)	0.0510 (16)	−0.0155 (13)	−0.0136 (13)	−0.0142 (14)
C10	0.0496 (18)	0.0541 (17)	0.0458 (16)	−0.0208 (15)	−0.0087 (13)	−0.0116 (14)
C11	0.063 (2)	0.059 (2)	0.066 (2)	−0.0120 (17)	−0.0275 (17)	−0.0062 (17)
C12	0.100 (4)	0.057 (2)	0.175 (5)	−0.034 (2)	−0.038 (4)	−0.019 (3)

### Geometric parameters (Å, °)

**Table:**

Br—C4	1.898 (3)	C4—C5	1.400 (5)
S—O2	1.493 (3)	C5—C6	1.374 (5)
S—C1	1.755 (3)	C5—H5	0.9300
S—C11	1.793 (4)	C6—C7	1.376 (4)
O1—C7	1.370 (3)	C6—H6	0.9300
O1—C8	1.375 (3)	C8—C9	1.481 (4)
O3—C10	1.327 (4)	C9—C10	1.501 (4)
O3—C12	1.454 (5)	C9—H9A	0.9700
O4—C10	1.195 (4)	C9—H9B	0.9700
C1—C8	1.350 (4)	C11—H11A	0.9600
C1—C2	1.451 (4)	C11—H11B	0.9600
C2—C3	1.391 (4)	C11—H11C	0.9600
C2—C7	1.395 (4)	C12—H12A	0.9600
C3—C4	1.377 (4)	C12—H12B	0.9600
C3—H3	0.9300	C12—H12C	0.9600
			
O2—S—C1	106.07 (14)	C6—C7—C2	123.2 (3)
O2—S—C11	106.21 (17)	C1—C8—O1	111.1 (2)
C1—S—C11	98.55 (16)	C1—C8—C9	133.3 (3)
C7—O1—C8	106.4 (2)	O1—C8—C9	115.6 (2)
C10—O3—C12	116.0 (3)	C8—C9—C10	114.0 (2)
C8—C1—C2	107.2 (3)	C8—C9—H9A	108.8
C8—C1—S	124.7 (2)	C10—C9—H9A	108.8
C2—C1—S	127.9 (2)	C8—C9—H9B	108.8
C3—C2—C7	120.0 (3)	C10—C9—H9B	108.8
C3—C2—C1	135.6 (3)	H9A—C9—H9B	107.7
C7—C2—C1	104.4 (2)	O4—C10—O3	123.4 (3)
C4—C3—C2	116.2 (3)	O4—C10—C9	126.2 (3)
C4—C3—H3	121.9	O3—C10—C9	110.4 (3)
C2—C3—H3	121.9	S—C11—H11A	109.5
C3—C4—C5	123.5 (3)	S—C11—H11B	109.5
C3—C4—Br	118.6 (3)	H11A—C11—H11B	109.5
C5—C4—Br	117.9 (2)	S—C11—H11C	109.5
C6—C5—C4	119.9 (3)	H11A—C11—H11C	109.5
C6—C5—H5	120.0	H11B—C11—H11C	109.5
C4—C5—H5	120.0	O3—C12—H12A	109.5
C5—C6—C7	117.1 (3)	O3—C12—H12B	109.5
C5—C6—H6	121.5	H12A—C12—H12B	109.5
C7—C6—H6	121.5	O3—C12—H12C	109.5
O1—C7—C6	125.9 (3)	H12A—C12—H12C	109.5
O1—C7—C2	110.9 (2)	H12B—C12—H12C	109.5
			
O2—S—C1—C8	131.5 (3)	C5—C6—C7—C2	−1.3 (5)
C11—S—C1—C8	−118.8 (3)	C3—C2—C7—O1	−177.8 (2)
O2—S—C1—C2	−42.4 (3)	C1—C2—C7—O1	1.1 (3)
C11—S—C1—C2	67.3 (3)	C3—C2—C7—C6	1.4 (4)
C8—C1—C2—C3	177.9 (3)	C1—C2—C7—C6	−179.7 (3)
S—C1—C2—C3	−7.3 (5)	C2—C1—C8—O1	0.1 (3)
C8—C1—C2—C7	−0.7 (3)	S—C1—C8—O1	−174.9 (2)
S—C1—C2—C7	174.1 (2)	C2—C1—C8—C9	−179.8 (3)
C7—C2—C3—C4	−0.3 (4)	S—C1—C8—C9	5.1 (5)
C1—C2—C3—C4	−178.8 (3)	C7—O1—C8—C1	0.6 (3)
C2—C3—C4—C5	−0.8 (4)	C7—O1—C8—C9	−179.5 (2)
C2—C3—C4—Br	178.5 (2)	C1—C8—C9—C10	63.6 (4)
C3—C4—C5—C6	0.9 (5)	O1—C8—C9—C10	−116.3 (3)
Br—C4—C5—C6	−178.4 (2)	C12—O3—C10—O4	0.8 (5)
C4—C5—C6—C7	0.1 (5)	C12—O3—C10—C9	−179.9 (4)
C8—O1—C7—C6	179.7 (3)	C8—C9—C10—O4	−12.9 (5)
C8—O1—C7—C2	−1.1 (3)	C8—C9—C10—O3	167.7 (3)
C5—C6—C7—O1	177.8 (3)		

### Hydrogen-bond geometry (Å, °)

**Table:**

*D*—H···*A*	*D*—H	H···*A*	*D*···*A*	*D*—H···*A*
C3—H3···O2^i^	0.93	2.43	3.334 (4)	163
C9—H9B···O1^ii^	0.97	2.59	3.555 (3)	171
C9—H9A···O2^iii^	0.97	2.22	3.177 (4)	169
C12—H12B···Cg^iv^	0.96	2.99	3.903 (4)	159

Symmetry codes: (i) −*x*+1, −*y*+1, −*z*; (ii) −*x*+2, −*y*+1, −*z*+1; (iii) −*x*+2, −*y*+1, −*z*; (iv) *x*, *y*+1, *z*.
